# Draft Genome Sequences of Five Brazilian *Clostridium botulinum* Group III Type D/C Strains

**DOI:** 10.1128/genomeA.00349-17

**Published:** 2017-05-18

**Authors:** Cédric Woudstra, Roseane B. Brito, Antônio A. Fonseca Júnior, Rodrigo O. S. Silva, Francisco C. F. Lobato, Patrick Fach

**Affiliations:** a ANSES, Food Safety Laboratory, Platform IdentyPath, Université Paris-Est, Maisons-Alfort, France; b Brazilian Ministry of Agriculture, Livestock and Food Supply (MAPA), National Agricultural Laboratory (LANAGRO/MG), Minas Gerais, Brazil; c Veterinary School, Universidade Federal de Minas Gerais (UFMG), Minas Gerais, Brazil

## Abstract

Animal botulism is mainly associated with *Clostridium botulinum* group III-producing neurotoxin types C, C/D, D, and D/C. In this report, we present the draft genome sequences of the first five strains of *Clostridium botulinum* type D/C isolated in Brazil and used for vaccination purposes.

## GENOME ANNOUNCEMENT

Animal botulism is caused by group III *Clostridium botulinum* strains that produce type C and D toxins, or a chimeric fusion of C and D termed C/D or D/C toxins (1). Animal botulism is considered an emerging disease in Europe, notably in poultry production (2), where it could lead to significant economic losses (3). It has been shown previously that animal botulism in Europe is mainly due to mosaic type C/D strains for avian species and type D/C strains for bovines (4). In Brazil, the disease is endemic in avian species and in domestic ruminants (5, 6), but the type of botulism is clouded by the absence of molecular studies with isolated strains.

Currently, the genome sequences of *Clostridium botulinum* group III strains available in the public database are limited to 2 type C, 21 type C/D, 3 type D, and 5 type D/C strains. They originate from different countries, but none originate from South America.

In order to investigate the epidemiological genetic relationship of strains originating from different geographical areas, we sequenced the genomes of five strains of *Clostridium botulinum* group III type D/C. They all originated from Brazil.

Genomic DNA was extracted from a 48-h culture, incubated at 37°C under anaerobic conditions in tryptone-peptone-glucose-yeast extract (TPGY) medium, using the DNeasy blood and tissue kit (Qiagen, Hilden, Germany), according to the manufacturer’s instructions for Gram-positive bacteria. Libraries were prepared using the Nextera XT kit (Illumina). Whole-genome sequencing was performed using an Illumina MiSeq platform (Illumina), according to the manufacturer’s instructions. Five MiSeq runs were carried out, three with paired-end 150-nucleotide (nt) reads on MiSeq version 2 nano, one with version 2 micro, and the last with version 2 standard chemistry. The raw reads were trimmed (minimum length, 35 bp; quality score, 0.03) and assembled in CLC Genomics Workbench 8.0.2 by *de novo* assembly (minimum contig length, 1,000 bp), producing 105 to 190 contigs (Table 1). The median read depth of the assemblies ranged from 30× for isolates 1275 and 1276 to 450× for isolate 1274, with *N*_50_ values between 34 kbp and 47 kbp (Table 1). The sequences were annotated with the National Center for Biotechnology Information (NCBI) Prokaryotic Genome Automatic Annotation Pipeline (PGAAP) (7).

**TABLE 1:** NCBI accession numbers and assembly metrics of *Clostridium botulinum* group III draft genome sequences

Isolate	Origin	*bont* type	No. of contigs	Genome size (Mbp)	G+C (%)	*N*_50_ (kbp)	Median read depth (×)	No. of coding sequences (per PGAAP)	NCBI accession no.	SRA accession no.
1274	Vaccine	D/C	122	2.94	28.0	43.278	450	2,648	MVIY00000000	SRR5239628
1275	Vaccine	D/C	131	2.94	27.9	40.516	30	2,642	MVIZ00000000	SRR5239627
1276	Vaccine	D/C	127	2.91	27.9	47.330	30	2,634	MVJA00000000	SRR5239626
1277	Vaccine	D/C	105	2.57	28.1	40.321	50	2,311	MVJB00000000	SRR5239625
CP05	Vaccine	D/C	190	2.88	28.0	34.348	200	2,537	MVJC00000000	SRR5239624

The average size of the genomes in this study is 2.94 Mb, with 2.57 Mb being the smallest genome size, an average G+C content of 28.1% (isolate 1277, Table 1), and 2.94 Mb as the largest genome size (isolate 1274, Table 1). On average, 2,554 coding sequences were identified in the genomes (Table 1). A preliminary phylogenetic analysis showed the sequences to be related to group Ib of the *C. botulinum* group III genomes, as defined previously (8). A detailed report on further analyses of the draft genome sequences will be released in a future publication.

### Accession number(s).

The annotated draft whole-genome sequences of these *Clostridium botulinum* group III strains were deposited in DDBJ/ENA/GenBank under the accession numbers listed in Table 1. The versions described in this paper are the first versions.
